# Alterations of plasma cytokine biomarkers for identifying age at onset of schizophrenia with neurological soft signs

**DOI:** 10.7150/ijms.38891

**Published:** 2020-01-14

**Authors:** Jia-Yun Liu, Han-Yu Chen, Jin-Jia Lin, Ming-Kun Lu, Hung-Pin Tan, Fong-Lin Jang, Sheng-Hsiang Lin

**Affiliations:** 1 Institute of Clinical Medicine, College of Medicine, National Cheng Kung University, Tainan, Taiwan; 2 Department of Medical Research, Center for Medical Genetics, Changhua Christian Hospital, Changhua, Taiwan; 3 Department of Genomic Medicine, Center for Medical Genetics, Changhua Christian Hospital, Changhua, Taiwan; 4 Department of Psychiatry, Chimei Medical Center, Tainan, Taiwan; 5 Department of Health, Jianan Mental Hospital, Tainan, Taiwan; 6 Department of Applied Life Science and Health, Chia Nan University of Pharmacy and Science, Tainan, Taiwan; 7 Department of Psychiatry, Kaohsiung Veterans General Hospital Tainan Branch, Tainan, Taiwan; 8 Department of Environmental and Occupational Health, College of Medicine, National Cheng Kung University, Tainan, Taiwan; 9 Department of Public Health, College of Medicine, National Cheng Kung University, Tainan, Taiwan; 10 Biostatistics Consulting Center, National Cheng Kung University Hospital, College of Medicine, National Cheng Kung University, Tainan, Taiwan

**Keywords:** schizophrenia, early-onset, immune dysregulation, neurodevelopment, discriminant analysis.

## Abstract

Several studies have been suggested that immunity plays a part in neurodevelopment and schizophrenia pathogenesis. Early age of onset in schizophrenia is associated with genetic factors which affect neurodevelopment. This study aims to identify immune abnormalities associated with neurodevelopmental impairments in early-onset schizophrenia (EOS) and adult-onset schizophrenia (AOS) patients. We determined the plasma levels of six cytokines (IL-1β, IL-4, IL-6, IL-10, IL-12 and TNF-α) in schizophrenia patients and healthy controls. Measurements included neurological soft signs (NSS) to distinguish and subgroup those with neurodevelopmental impairments. The study included 210 schizophrenia patients, which were divided into 84 EOS and 126 AOS patients, as well as 122 healthy controls. We observed significant differences in levels of IL-4, IL-6 and IL-10 between EOS and AOS patients. The results demonstrated the area under ROC curve (AUC) of the IL-4 in EOS and healthy controls was 0.81. Moreover, these results indicated that AUC of the IL-4 and the combination of IL-4, IL-6 and IL-12 in EOS with NSS and healthy controls were 0.91 and 0.95. These cytokines are altered in EOS and schizophrenia patients with neurodevelopmental impairments and demonstrated good classification abilities. These findings manifested that both pro- and anti-inflammatory cytokines are contributed to the clinical and pathophysiological features of schizophrenia. Future works are expected to explore potential genetic effectors and predictors as well as therapeutic directions in personalized medicine for early-onset schizophrenia.

## Introduction

Various studies indicated that psychosis is closely linked to immune responses 1-4. A possible association between schizophrenia and immune system is supported by epidemiological and genetic studies pointing to links with infection and inflammation 5, 6. In the past, the brain has been considered an area restricted from immunity, shielded by the blood-brain barrier (BBB), but some research proposes an alternative perspective 7. In response to systemic inflammation, microglia release inflammatory mediators such as cytokines, which reflect brain injury and would alter neurodevelopment, cognition, and mood 8. Some review articles have provided evidences of altered levels of cytokines in patients with schizophrenia, such as increased levels of interleukin (IL)-1, IL-6, and tumor necrosis factor-α (TNF-α) 9, 10. Other studies have also demonstrated an imbalance between T helper 1 (Th1) and T helper 2 (Th2) cytokines in schizophrenia 11, 12. Thus, the inflammatory cytokines involved in adaptive immunity likely plays an important role in the pathophysiology of schizophrenia.

The neurodevelopmental model of schizophrenia posits that environmental influences or genetic factors might initiate pathological processes before disease onset 13, 14. Abnormal neural circuit or brain region development in late adolescence may lead to an onset of psychosis 15, 16. Previous studies have shown that more developmental impairments are present in children who are at a high risk for schizophrenia, such as those with impairments in neuromotor, attention, declarative memory or psychomotor function 17. The neurodevelopmental hypothesis demonstrates the possibility of a connection with faulty brain development in schizophrenia. Previous studies have reported that early-onset schizophrenia (EOS) patients in particular present with more severe symptoms, additionally they are more treatment-resistant and have poorer prognosis than adult-onset schizophrenia (AOS) patients 14. In Taiwan, the annual prevalence rate of schizophrenia patients with EOS was 41.8 per 100,000 persons among the youth population and the average annual incidence rate was 15.1 per 100,000 persons 18. Genetic factors appear to determine the age of onset and are the key factors in the prognosis of schizophrenia patients. An earlier age of onset is associated with a broad range of genetic factors and neurodevelopmental progressions. Based on these studies, it is evident that immune abnormalities are associated with schizophrenia, and inflammatory cytokines are critical factors in the disruption of neuron-glia interactions and resulting neurodevelopmental impairments.

Despite these findings, it remains unclear whether specific immunological alterations in patients with neurodevelopmental disabilities are linked to a genetic vulnerability to EOS. Communications between the immune system and brain could contribute to the impaired neurodevelopment, and neurodegenerative symptoms observed in schizophrenia patients 19. A genetic imbalance in immune responses has been identified, although the genomic instability of mechanisms linked to the immune disturbances apparent in schizophrenia remains to be elucidated. Therefore, we hypothesize that both pro-inflammatory and anti-inflammatory cytokines are associated with neurodevelopmental impairments in EOS and AOS patients. We intend to assess the association of adaptive immune imbalances, for example in Th1, Th2, or naïve T cell differentiation-related cytokines in schizophrenia patients with different ages at onset. Our specific aims are: (1) to examine the role of immune abnormalities in schizophrenia patients at different ages at onset; and (2) to explore the association of immune abnormalities with neurodevelopmental impairments in EOS and AOS patients.

## Methods

### Participants

All schizophrenia patients were recruited between August 2011 and March 2016 from Chi Mei Medical Center, Jianan Mental Hospital, and Lok An Hospital in Taiwan. Patients were enrolled based on the criteria for schizophrenia in the Diagnostic and Statistical Manual of Mental Disorders, Fourth Edition (DSM-IV). The Chinese version of the Diagnostic Interview for genetic studies (DIGS-C), including positive and negative symptom scales, as well as neurodevelopmental evaluations were conducted with all participants. Positive symptom severity in this study was assessed using the scale for the assessment of positive symptoms (SAPS) and negative symptom severity was assessed using the scale for the assessment of negative symptoms (SANS). In this study, we recruited 210 chronic schizophrenia patients with different onset ages and 122 healthy individuals to serve as controls (HC) between 20 and 65 years old. The patients with chronic schizophrenia met the following criteria: (1) onset of psychotic symptoms more than 24 months earlier; and (2) currently receiving antipsychotic medication treatment. The healthy controls met the criteria: (1) absence of a current or past psychiatric disorder as confirmed by DIGS-C. There were 84 patients with early-onset schizophrenia (EOS, onset age < 20), 126 patients with adult-onset schizophrenia (AOS, onset age ≥ 20) for the subsequent analyses. The age of schizophrenia onset was defined via consulting patients' family members and tracking the medical records. Patients with other major psychiatric disorders, severe neurological abnormalities, mental retardation, substance-related disorders, somatic disorders with neurological components, traumatic brain injuries, or prominent drug use problems were excluded from this study. The healthy controls with a personal or family history of psychiatric disorder were also excluded from the control group. This study's protocol, including its purpose, methods, and use of data collected, was approved by the institutional review board (IRB) of each participating hospital. All participants provided written informed consent to participate by signing the IRB-approved consent form.

### Plasma cytokines measurements

In order to determine the immune cytokines levels, we collected 10 ml of venous blood in VACUETTE® tubes containing EDTA from schizophrenia patients and healthy controls. Plasma samples were obtained by centrifugation at 3500 rpm (4℃) for 10 minutes and were stored at -80℃ until assay. Levels of six plasma cytokines (IL-1β, IL-4, IL-6, IL-10, IL-12 and TNF-α) were measured using a sandwich enzyme-linked immunosorbent assay (ELISA) (eBioscience Inc., San Diego, CA, USA) according to the manufacturer's instructions. Samples from different groups were assayed on the same ELISA plate to minimize experimental variation.

### Assessment of neurological soft signs

Neurological soft signs (NSS) are minor neurological abnormalities. The NSS scores were evaluated according to the NES Scale by Buchanan and Heinrichs (1989). A total of 26 items are assigned using four subscales according to dysfunction in sensory integration, motor coordination, motor sequencing, or other 20, 21. Sensory integration includes assessments of audiovisual integration, stereognosis, graphesthesia, extinction, and confusion of right and left; motor coordination includes assessments of tandem walking, rapid alternating movements, finger-thumb opposition, and the finger-nose test; motor sequencing includes the fist-ring, fist-edge-palm, Ozeretski, and rhythm tapping tests; others include assessing adventitious overflow, the Romberg test, tremors, testing memory (5 or 10 min), a rhythm tapping test, assessing mirror movements, synkinesis, convergence, and gaze impersistence. The items were scored on a three-point scale: 0 indicates no abnormality, 1 indicates mild but definite abnormality, and 2 marks impairment. The patients with NSS impairments were defined as patients' score more than third quartile of the total NSS score. The trained raters were blind to the subgroups of the schizophrenia patients. However, they were not blind to the allocation of the patients and healthy controls. The NSS scale reliability evaluation and standardized measurement was established by a neurologist in the Chi Mei Medical Center to allow sufficient internal and inter-rater reliability.

### Statistical analysis

All analyses were conducted in SAS version 9.4 (SAS Institute Inc) and blinded to the study groups. The results were reported as the mean and standard deviation (SD). For categorical variables, comparisons between groups were performed using Pearson's chi-square test or Fisher exact test to determine the significant differences between expected and observed frequencies in one or more categories. In addition, Student's t-test was used for continuous variables. A p-value of less than 0.05 was considered statistically significant.

The analysis of the receiver operating characteristic (ROC) curves was conducted using logistic regression to plot the accuracy, sensitivity and specificity with multivariate cytokine levels in schizophrenia patients and healthy controls 22. The area under each curve (AUC) was calculated as the predictive value of using cytokine levels to accurately differentiate between schizophrenia patients and healthy controls based on the cytokine levels found in our samples.

We also applied a partial least squares-linear discriminant analysis (PLS-LDA) to perform multivariate analyses for group classification by using plasma cytokines as independent variables to further distinguish the study groups 23, 24. Variable values were derived from the PLS model regression coefficients in an attempt to express one dependent variable as a linear combination of other features or measurements. Two-dimensional score plots were produced to visually assess the distance between groups. Variable importance for projection (VIP) scores were used to identify the most influential features in the PLS-LDA model 25. The values of the PROC PLS components were then transferred to PROC DISCRIM for classification. The results of discriminant analysis were summarized in terms of each group's correct classification accuracy and cross-validation accuracy.

## Results

### Demographic data

Patient demographic characteristics are summarized in Table 1. We recruited 210 schizophrenia patients and 122 healthy individuals as controls (HC) in this study. There were 84 early-onset schizophrenia (EOS) patients, whose age of onset was before their 20th birthday and 126 adult-onset schizophrenia (AOS) patients whose onset age were at least 20 years old. EOS patients (N=84), AOS patients (N=126), and healthy controls (N=122) were compared in terms of sex, age, smoking, drinking, BMI, education and NSS scale as well as positive and negative scale of DIGS-C. There were significant differences in sex, smoking and education between patients and controls. The mean onset ages of EOS patients and AOS patients were 16.7 and 27 years old, respectively. There were significant differences in the presence of NSS scale between the two groups of schizophrenia patients and the HC group. In addition, both positive and negative DIGS-C scales (SAPS and SANS, respectively) were statistically significant in EOS and AOS patients (p < 0.05). Furthermore, we presented the data separately for males and females in Table S1. There was no significant difference of most characteristics between males and females in the study groups.

### Cytokine levels in schizophrenia patients and controls

We determined the plasma levels of cytokines in schizophrenia patients and healthy controls (Table 2). In EOS patients and healthy controls, only the levels of IL-1β were not significantly different. The cytokines levels except IL-1β and IL-4 were significantly different in the AOS patients compared to the controls although there was a trend towards lower IL-4 levels in AOS group compared to the control group. In addition, AOS patients and healthy controls did not significantly differ in IL-1β and IL-4 levels, both these two cytokines levels were still lower in control group. On the other hand, the levels of IL-4, IL-6 and IL-10 were significantly different between EOS and AOS patients. These results indicated that these pro-inflammatory and anti-inflammatory cytokine levels in schizophrenia patients significantly differed from healthy controls and additionally we observed differences in cytokines levels between EOS and AOS patients.

We further evaluated whether the severity of schizophrenia in all patients as well as early-onset and adult-onset would affect the cytokines levels. We determined the plasma levels of cytokines in all schizophrenia patients and even divided them into subgroups based on the median of the summary SAPS and SANS score (Table 3). The results are shown in supplementary materials (Table S2 and S3) and indicated that there was no significant difference between each subgroup in EOS and AOS patients.

### Classification efficiency of cytokines in schizophrenia patients and controls

Logistic regression analysis was used to evaluate the classification efficiency of differential diagnoses of schizophrenia patients and healthy controls. AUCs of ROC curves were calculated to evaluate the performance of the classifier. Table 4 showed the ROC curves for the plasma cytokines in schizophrenia, EOS and AOS patients versus healthy controls. The results of the ROC analysis of the most significant cytokine, IL-4, was 0.71 (95% CI = 0.65 - 0.76), indicating good performance using the single plasma cytokine. The AUCs for the combined cytokines (IL-4, IL-6 and IL-12) was 0.75 (95% CI = 0.69 - 0.80) between schizophrenia patients and healthy controls. The ROC curves of the plasma cytokines also correctly identified patients with EOS patients and healthy controls, the AUC of IL-4 was 0.81 (95% CI = 0.74 - 0.88) which showed the enough discriminant ability. Additionally, the AUC of the cytokine's combination (IL-4, IL-6 and IL-12) was 0.83 (95% CI = 0.77 - 0.90) (Figure S1). On the other hand, the AUC of IL-4 was 0.64 (95% CI = 0.57 - 0.71) between AOS patients and healthy controls. Furthermore, the AUC result for the combined IL-4, IL-6 and IL-12 was 0.70 (95% CI = 0.64 - 0.77). In summary, the most significant result of using single marker from the ROC analysis was IL-4 which indicated that a single plasma cytokine had an enough performance than multiple plasma cytokines in EOS patients and healthy controls.

### Classification efficiency of cytokines in schizophrenia and EOS patients with neurodevelopmental impairments

Table 5 displayed the results of ROC analysis for schizophrenia or EOS patients with neurological soft signs (NSS) versus healthy controls. We analyzed all of the six cytokines respectively and found the significant influence of IL-4. The plasma levels of IL-4 indicated a good ability to classify schizophrenia patients with NSS versus healthy controls (AUC = 0.86). Additionally, the combined cytokines, IL-4, IL-6 and IL-12, showed a significant classification (AUC = 0.89). Moreover, the results demonstrated the discriminatory ability of IL-4 in EOS patients with NSS versus healthy controls (AUC = 0.91) and the combination of IL-4, IL-6 and IL-12 was result in a significant discriminant (AUC = 0.95). On the other hand, the results showed the discriminant performance of IL-4 in AOS patients (AUC = 0.74) and healthy controls, as well as the same result in combined cytokines (IL-4, IL-6 and IL-12). These results indicate a better ROC curve analysis result for EOS patients and healthy controls with the highest AUC of discrimination (Figure S2).

### Discriminant analysis of schizophrenia, EOS and AOS patients with neurodevelopment impairments

**Figure 1:**
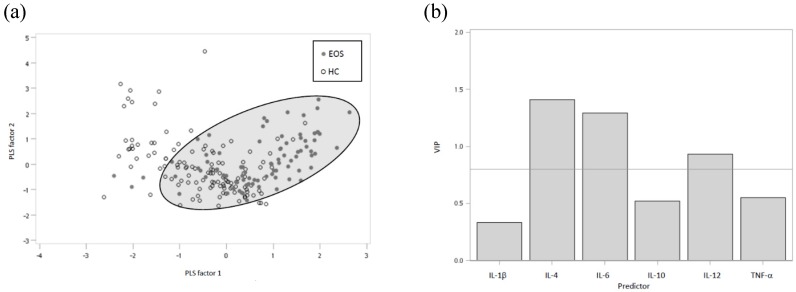
(a) Score plot derived from partial least squares-linear discriminant analysis (PLS-LDA) based on the 6 plasma cytokines levels. (b) Variable importance in the projection (VIP) data of the compounds with VIP values in EOS patients and healthy controls.

The Partial least squares-linear discriminant analysis was performed to identify differences between the groups, with the best class functions showing clear separations by partial-least-squares (PLS) factors (Figure 1a). The partial-least-squares linear discriminant analysis (PLS-LDA) score plot illustrates a nearly perfect separation of EOS patients and healthy controls. For the PLS-LDA model, variable importance in projection (VIP) values were calculated and used to identify important variables (Figure 1b). A VIP greater than 0.8 identified the more influential cytokines for each PLS-LDA plot. We applied the linear discriminant analysis (LDA) to determine the linear combination of variables to classify schizophrenia, EOS and AOS patients as well as healthy controls (Table 4) and to understand how these variables are related to each other to determine compliance. The effective classification accuracy of the combination (IL-4, IL-6 and IL-12) was 77% and the cross-validation accuracy was 76% in schizophrenia patients. Furthermore, the accuracy of the combined IL-4, IL-6 and IL-12 was 80% and the cross-validation accuracy was 79% in EOS patients. On the other hand, the discriminant results of same combination in AOS patients were 77% and 75% respectively.

We further conducted the discriminant function in schizophrenia, EOS and AOS patients with NSS versus healthy controls. The classification accuracy of combined cytokines, IL-4, IL-6 and IL-12, was 79% and cross-validation accuracy was 79% for schizophrenia patients with NSS compared with healthy controls (Table 5). Additionally, the discriminant accuracy for EOS patients was 84% and the validation accuracy was 84%. On the other hand, the accuracy of same combination for AOS patients was 69% and the validation accuracy was 57%. These results indicate a higher correct discriminant classification accuracy in schizophrenia patients, even in EOS patients with NSS, compared to healthy controls.

## Discussion

This study highlights the association between immune cytokines and neurodevelopmental markers for schizophrenia patients. These cytokines involved in peripheral immune system-brain communication are thought to play roles in schizophrenia pathogenesis. Neurological soft signs have been consistently reported as endophenotypes in patients with schizophrenia and their relatives 26. EOS patients with neurodevelopmental impairments show significant abnormalities in plasma IL-4 levels compared with patients without neurodevelopmental impairments. IL-4 may contribute to brain repair through modulating microglia and macrophage function, as well as reduce neuronal damage with its pro-oxidative effects 27. From these results, we suggest that IL-4 might be involved in neurodevelopment and associated with EOS patients. More evidence of a correlation between inflammatory cytokines and neurodevelopmental markers is suggested to carry out these studies in the future. Among the cytokines analyses in the peripheral blood, we found that IL-4 levels were more discriminatory than others, though comparison of EOS patients and healthy controls had a higher AUC than for schizophrenia patients and AOS patients. On the other hand, our study was also focused on the relationship between neurodevelopmental and inflammatory pathways. Neurodevelopmental impairments in EOS patients were slightly different from AOS patients, which is consistent with the hypothesis of immune abnormality being associated with neurodevelopmental impairments in different schizophrenia patients according to age of onset.

Levels of IL-1β, IL-4, IL-6, IL-10, IL-12 and TNF-α have previously been studied in a meta-analysis 3. The levels of IL-1β and TNF-α were significantly higher in schizophrenia patients than in the control group, and there were no significant differences observed between medicated and unmedicated patients 28. Previous studies have also reported that plasma IL-4 concentration is lower in unmedicated schizophrenia patients 11. The findings of this study suggest that abnormalities in plasma cytokine levels are directly or indirectly related to the disorder, as opposed to the medication. Comparing chronic schizophrenia patients compared with the control group, we observed a large effect on IL-1β, IL-6, and TNF-α levels. Furthermore, the IL-12 concentrations were decreased in both EOS and AOS patients compared to healthy controls, the results were consistent to the previous evidence that antipsychotic drugs may suppress IL-12 concentration in psychiatric patients 29. Elevated IL-6 levels in childhood might lead to the development of schizophrenia in young adulthood 30, and significant correlations have been found between anti-inflammatory IL-4 and IL-10 with negative symptoms in EOS patients 31.

There are several limitations of our study. First, this study was cross-sectional, and we can't determine the course of immune markers from the time of psychosis onset to later phases of schizophrenia. Longitudinal studies are required to determine cytokines dysregulations in schizophrenia across the disease duration. In previous studies, IL-1β and IL-4 levels did not change during antipsychotic treatment 32, 33. We also collected the medical records from the patients and evaluated the influence of cytokines due to antipsychotic medication. There were no significant differences in the chronic schizophrenia patients with various antipsychotic medication in our study. Our results were consistent to previously mentioned studies that altered peripheral cytokine levels do not necessarily correlate in chronically ill patients receiving antipsychotic treatment. 32-34 Nevertheless, the biological and clinical significance of antipsychotic drugs is largely unclear and remains to be elucidated; further studies with unmedicated schizophrenia patients are necessary. Second, the study was restricted to analyzing the cytokines selected. Multiplex assays may be used to assess more cytokines involved in immune system alterations.

## Conclusions

In conclusion, our results provide evidence of lower plasma IL-4 levels in EOS patients compared with healthy controls. Plasma IL-4 levels were also significantly different between EOS patients with and those without neurodevelopmental impairments. In summary, the findings may support the use of cytokines as potential biomarkers to identify EOS or AOS in patients. Alteration in IL-4 levels in schizophrenia patients is reportedly linked to neurodevelopmental outcomes with immune dysregulation. This study demonstrated that the discriminatory abilities of cytokines are sufficient to identify EOS patients with neurodevelopmental impairments, which helps explain some of the clinical and pathophysiological features of schizophrenia. Future studies may seek to clarify potential biomarkers and therapeutic strategies for personalized medicine in EOS patients by incorporating information about molecular mechanisms and genetic determinants.

## Supplementary Material

Supplementary figures and tables.

## Figures and Tables

**Table 1:** Characteristics of schizophrenia patients and healthy controls.

	Schizophrenia Patients		Healthy Controls
Characteristics	EOS (N=84)	AOS (N=126)	(N=122)
	N (%)	N (%)	N (%)
Male	57^a^ (67.9)	85^b^ (67.5)	46 (37.7)
Drinking	5 (6.0)	13 (10.3)	17 (13.9)
Smoking	29^b^ (34.5)	62^b,c^ (49.6)	16 (13.1)
	Mean (SD)	Mean (SD)	Mean (SD)
Age	38^a^ (9)	44^c^ (9)	41.1 (11.8)
Onset age	16.7 (1.9)	27 (6.4)	-
BMI	25.9 (5.1)	26.1^b^ (5.4)	24.8 (4.5)
Disease duration	20.8 (8.8)	16.4 (9.5)	-
Education	11.4^a^ (3.1)	11.7^b^ (2.9)	14.1 (3.4)
NSS	10.3^a^ (7.0)	7.3^b^ (4.9)	1.6 (1.7)
SAPS score	51.67 (30.84)	39.73^c^ (27.68)	-
SANS score	62.26 (28.63)	51.58^c^ (24.14)	-
Summary SAPS and SANS score	113.93 (55.68)	90.77^c^ (42.66)	-

EOS, early-onset schizophrenia; AOS, adult-onset schizophrenia; BMI, body mass index; NSS, neurological soft signs; SAPS, scale for assessment of positive symptoms; SANS, scale for assessment of negative symptoms. ^a^ significant difference between EOS and healthy controls, p < 0.05. ^b^ significant difference between AOS and healthy controls, p < 0.05. ^c^ significant difference between EOS and AOS patients, p < 0.05.

**Table 2:** Comparison of plasma cytokines in patients with early-onset and adult-onset schizophrenia versus healthy controls.

	EOS	AOS	HC		P-value	
Cytokines	(N=84)	(N=126)	(N=122)	EOS vs. HC	AOS vs. HC	EOS vs. AOS
	Mean (SD)	Mean (SD)	Mean (SD)			
IL-1β	1.22 (0.76)	1.20 (0.84)	1.59 (3.13)	0.2	0.2	0.4
IL-4	1.50 (2.31)	2.67 (2.80)	3.24 (2.00)	< 0.0001	0.07	0.0004
IL-6	2.01 (1.48)	1.67 (1.75)	1.17 (0.93)	< 0.0001	0.005	0.03
IL-10	2.78 (0.73)	2.53 (1.00)	3.17 (1.89)	0.04	0.001	0.02
IL-12	1.07 (0.60)	1.06 (0.68)	1.56 (1.16)	< 0.0001	< 0.0001	0.4
TNF-α	14.14 (14.05)	12.91 (14.77)	20.84 (31.26)	0.04	0.01	0.5

EOS, early-onset schizophrenia; AOS, adult-onset schizophrenia; HC, healthy controls.

**Table 3:** Stratified comparison of summary SAPS and SANS score-based schizophrenia subgroups with plasma cytokines in early-onset and adult-onset schizophrenia patients.

	EOS (N=84)			AOS (N=126)		
	Low	High		Low	High	
Cytokines	Mean (SD)	Mean (SD)	P value	Mean (SD)	Mean (SD)	P value
IL-1β	1.14 (0.72)	1.37 (0.82)	0.17	1.18 (0.86)	1.15 (0.84)	0.93
IL-4	1.35 (2.30)	1.77 (2.34)	0.11	2.59 (2.72)	2.87 (3.09)	0.87
IL-6	2.10 (1.38)	1.87 (1.67)	0.39	1.64 (1.65)	1.75 (2.01)	0.93
IL-10	2.66 (0.65)	2.98 (0.83)	0.12	2.51 (0.98)	2.56 (1.12)	0.90
IL-12	1.03 (0.64)	1.13 (0.54)	0.47	1.05 (0.68)	1.08 (0.71)	0.99
TNF-α	15.30 (14.45)	12.06 (13.29)	0.24	14.58 (16.43)	8.56 (7.78)	0.18

SZ, schizophrenia; EOS, early-onset schizophrenia; AOS, adult-onset schizophrenia; SAPS, scale for assessment of positive symptoms; SANS, scale for assessment of negative symptoms. High and low score subgroups were partitioned from the schizophrenia patients based on the median of summary SAPS and SANS score.

**Table 4:** Results of ROC curves and partial least squares-linear discriminant analysis in schizophrenia, EOS and AOS patients versus healthy controls.

	ROC curves analysis			PLS-LDA	
	AUC (95% CI)	Sensitivity	Specificity	Accuracy (%)	Cross-validation accuracy (%)
Schizophrenia patients					
IL-4	0.71 (0.65, 0.76)	0.66	0.57	75	74
IL-6	0.59 (0.53, 0.65)	0.63	0.55	63	63
IL-12	0.66 (0.60, 0.73)	0.70	0.59	57	57
IL-4 + IL-6 + IL-12	0.75 (0.69, 0.80)	0.72	0.65	77	76
IL-1β + IL-4 + IL-6 + IL-10 + IL-12 + TNF-α	0.75 (0.70, 0.80)	0.72	0.65	74	73
EOS patients					
IL-4	0.81 (0.74, 0.88)	0.77	0.76	66	66
IL-6	0.67 (0.59, 0.75)	0.66	0.63	69	69
IL-12	0.66 (0.58, 0.73)	0.64	0.62	58	57
IL-4 + IL-6 + IL-12	0.83 (0.77, 0.90)	0.79	0.77	80	79
IL-1β + IL-4 + IL-6 + IL-10 + IL-12 + TNF-α	0.83 (0.77, 0.89)	0.80	0.71	82	78
AOS patients					
IL-4	0.64 (0.57-0.71)	0.61	0.50	70	70
IL-6	0.54 (0.46, 0.62)	0.56	0.55	61	61
IL-12	0.67 (0.60, 0.74)	0.67	0.64	57	57
IL-4 + IL-6 + IL-12	0.70 (0.64, 0.77)	0.67	0.64	77	75
IL-1β + IL-4 + IL-6 + IL-10 + IL-12 + TNF-α	0.72 (0.66, 0.78)	0.66	0.62	72	71

EOS, early-onset schizophrenia; AOS, adult-onset schizophrenia; CI, confidence interval; ROC, receiver operating characteristic curve; PLS-LDA, Partial least squares-linear discriminant analysis.

**Table 5:** Results of ROC curves and partial least squares-linear discriminant analysis in schizophrenia, EOS and AOS patients with neurological soft signs versus healthy controls.

	ROC curves analysis			PLS-LDA	
	AUC (95% CI)	Sensitivity	Specificity	Accuracy (%)	Cross-validation accuracy (%)
Schizophrenia patients with NSS					
IL-4	0.86 (0.77, 0.95)	0.83	0.79	69	69
IL-6	0.57 (0.43, 0.71)	0.52	0.48	64	64
IL-12	0.71 (0.60, 0.82)	0.69	0.67	63	60
IL-4 + IL-6 + IL-12	0.89 (0.80, 0.98)	0.83	0.79	79	79
IL-1β + IL-4 + IL-6 + IL-10 + IL-12 + TNF-α	0.88 (0.79, 0.97)	0.83	0.75	77	69
EOS patients with NSS					
IL-4	0.91 (0.83, 0.99)	0.90	0.82	79	79
IL-6	0.62 (0.46, 0.78)	0.55	0.57	67	67
IL-12	0.74 (0.64, 0.84)	0.75	0.61	71	71
IL-4 + IL-6 + IL-12	0.95 (0.89, 1.00)	0.95	0.84	84	84
IL-1β + IL-4 + IL-6 + IL-10 + IL-12 + TNF-α	0.95 (0.88, 1.00)	0.90	0.80	92	84
AOS patients with NSS					
IL-4	0.74 (0.53, 0.95)	0.67	0.62	64	64
IL-6	0.54 (0.20, 0.72)	0.56	0.59	53	48
IL-12	0.64 (0.37, 0.90)	0.67	0.53	59	59
IL-4 + IL-6 + IL-12	0.74 (0.51, 0.97)	0.78	0.54	69	57
IL-1β + IL-4 + IL-6 + IL-10 + IL-12 + TNF-α	0.74 (0.51, 0.97)	0.78	0.52	79	46

EOS, early-onset schizophrenia; CI, confidence interval; NSS, neurological soft signs; PLS-LDA, Partial least squares-linear discriminant analysis; ROC, receiver operating characteristic curve.
