# Supplementary material for: Alterations of plasma cytokine biomarkers for identifying age at onset of schizophrenia with neurological soft signs
Source: Int J Med Sci. 2020 Jan 14;17(2):255–62. doi: 10.7150/ijms.38891 (PMC6990880; doi:10.7150/ijms.38891)
Supplement: Supplementary file 1 — Supplementary figures and tables. [file ijmsv17p0255s1.pdf]

Table S1. Characteristics of schizophrenia patients and healthy controls.

Characteristics	Schizophrenia Patients				Healthy Controls (N=122)	
	EOS (N=84)		AOS (N=126)			
	Male (n=57)	Female (n=27)	Male (n=85)	Female (n=41)	Male (n=46)	Female (n=76)
	N (%)		N (%)		N (%)	
Drinking	4 (7.0)	1 (3.7)	13[b] (15.3)	0	12[c] (26.1)	5 (6.6)
Smoking	26[a] (45.6)	3 (11.1)	60[b] (71.4)	2 (4.9)	12[c] (26.1)	4 (5.3)
	Mean (SD)		Mean (SD)		Mean (SD)	
Age	37 (9)	38 (9)	44 (9)	42 (9)	38[c] (13)	43 (11)
Onset age	16.9 (1.9)	16.3 (1.9)	27.4 (6.0)	26.4 (7.1)	-	-
BMI	26.0 (5.6)	25.6 (3.7)	25.8 (5.2)	26.8 (5.8)	25.9[c] (3.8)	24.0 (4.8)
Disease duration	20.3 (9.0)	21.8 (8.6)	16.6 (9.8)	16.1 (8.9)	-	-
Education	11.0 (3.4)	12.1 (2.3)	11.4 (3.0)	12.4 (2.7)	15.2[c] (3.8)	13.5 (3.0)
NSS	10.8 (7.1)	9.3 (6.7)	7.3 (4.6)	7.3 (5.6)	1.6 (1.6)	1.6 (1.7)
SAPS score	51.75 (34.30)	48.60 (37.11)	43.51 (29.31)	32.26 (35.90)	-	-
SANS score	66.17 (28.73)	55.00 (31.43)	49.94 (29.18)	53.82 (31.84)	-	-
Summary SAPS and SANS score	120.07 (55.79)	103.60 (62.98)	92.88 (48.47)	87.74 (58.26)	-	-

EOS, early-onset schizophrenia; AOS, adult-onset schizophrenia; BMI, body mass index; NSS, neurological soft signs; SAPS, scale for assessment of positive symptoms; SANS, scale for assessment of negative symptoms.

[a] significant difference between males and females in EOS, $p < 0.05$.

[b] significant difference between males and females in AOS, $p < 0.05$.

[c] significant difference between males and females in healthy controls, $p < 0.05$.

Table S2. Stratified comparison of SAPS score-based schizophrenia subgroups with plasma cytokines in early-onset and adult-onset schizophrenia patients.

Cytokines	SZ (N=210)			EOS (N=84)			AOS (N=126)		
	Low	High		Low	High		Low	High	
	Mean (SD)	Mean (SD)	P value	Mean (SD)	Mean (SD)	P value	Mean (SD)	Mean (SD)	P value
IL-1β	1.24 (0.85)	1.09 (0.70)	0.46	1.17 (0.74)	1.33 (0.80)	0.28	1.28 (0.92)	0.91 (0.57)	0.07
IL-4	2.12 (2.64)	2.41 (2.78)	0.67	1.36 (2.26)	1.80 (2.44)	0.20	2.59 (2.77)	2.87 (2.97)	0.97
IL-6	1.68 (1.53)	2.10 (1.88)	0.19	1.94 (1.43)	2.16 (1.61)	0.60	1.52 (1.58)	2.06 (2.09)	0.29
IL-10	2.59 (0.88)	2.71 (1.01)	0.39	2.70 (0.67)	2.95 (0.82)	0.18	2.53 (0.99)	2.52 (1.10)	0.96
IL-12	1.09 (0.68)	1.00 (0.57)	0.45	1.08 (0.64)	1.04 (0.53)	0.74	1.09 (0.71)	0.97 (0.60)	0.40
TNF-α	14.00 (15.23)	12.02 (12.51)	0.45	14.35 (13.94)	13.70 (14.54)	0.69	13.77 (16.06)	10.75 (10.79)	0.50

SZ, schizophrenia; EOS, early-onset schizophrenia; AOS, adult-onset schizophrenia.

High and low score subgroups were partitioned from the schizophrenia patients based on the median of SAPS score.

Table S3. Stratified comparison of SANS score-based schizophrenia subgroups with plasma cytokines in early-onset and adult-onset schizophrenia patients.

	SZ (N=210)			EOS (N=84)			AOS (N=126)		
Cytokines	Low	High		Low	High		Low	High	
	Mean (SD)	Mean (SD)	P value	Mean (SD)	Mean (SD)	P value	Mean (SD)	Mean (SD)	P value
IL-1β	1.17 (0.81)	1.25 (0.83)	0.40	1.14 (0.73)	1.36 (0.80)	0.15	1.19 (0.85)	1.15 (0.85)	0.77
IL-4	2.08 (2.62)	2.49 (2.82)	0.31	2.30 (2.27)	1.87 (2.37)	0.08	2.53 (2.71)	3.04 (3.10)	0.57
IL-6	1.85 (1.64)	1.71 (1.69)	0.44	2.06 (1.34)	1.94 (1.73)	0.56	1.73 (1.79)	1.50 (1.65)	0.48
IL-10	2.57 (0.88)	2.76 (0.99)	0.21	2.70 (0.67)	2.91 (0.82)	0.39	2.49 (0.98)	2.63 (1.11)	0.51
IL-12	1.05 (0.67)	1.08 (0.60)	0.63	1.00 (0.64)	1.18 (0.51)	0.15	1.08 (0.69)	0.99 (0.66)	0.43
TNF-α	14.34 (15.64)	11.26 (11.16)	0.35	14.44 (14.50)	13.61 (13.43)	0.78	14.29 (16.35)	9.19 (8.36)	0.29

SZ, schizophrenia; EOS, early-onset schizophrenia; AOS, adult-onset schizophrenia.

High and low score subgroups were partitioned from the schizophrenia patients based on the median of SANS score.

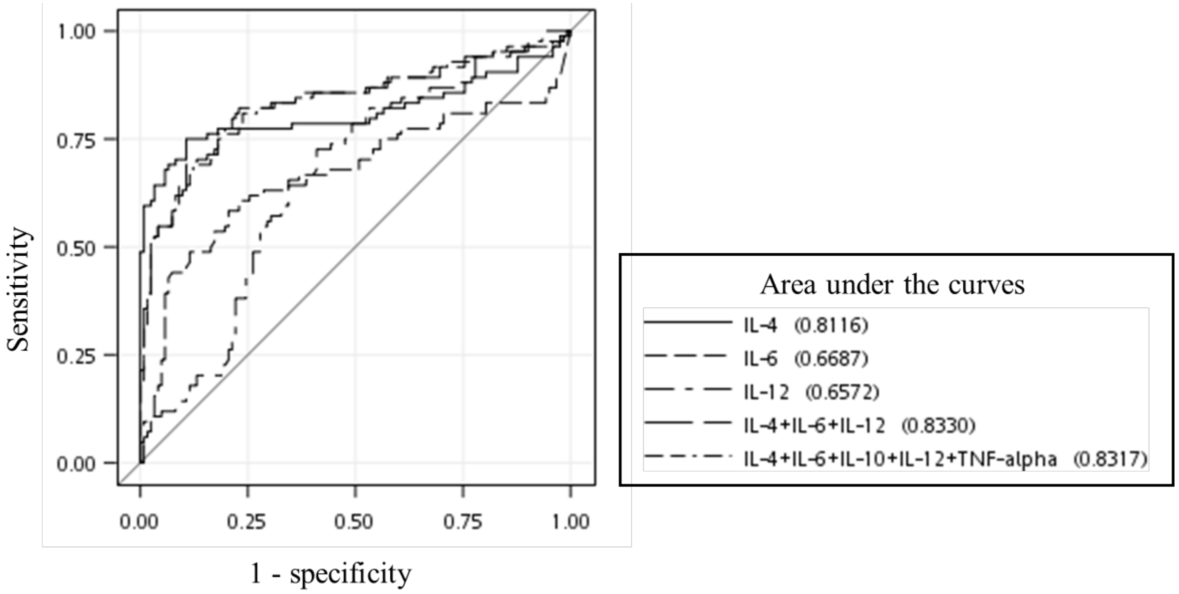

Figure S1. The receiver-operator characteristic (ROC) curves of plasma cytokines in early-onset schizophrenia.

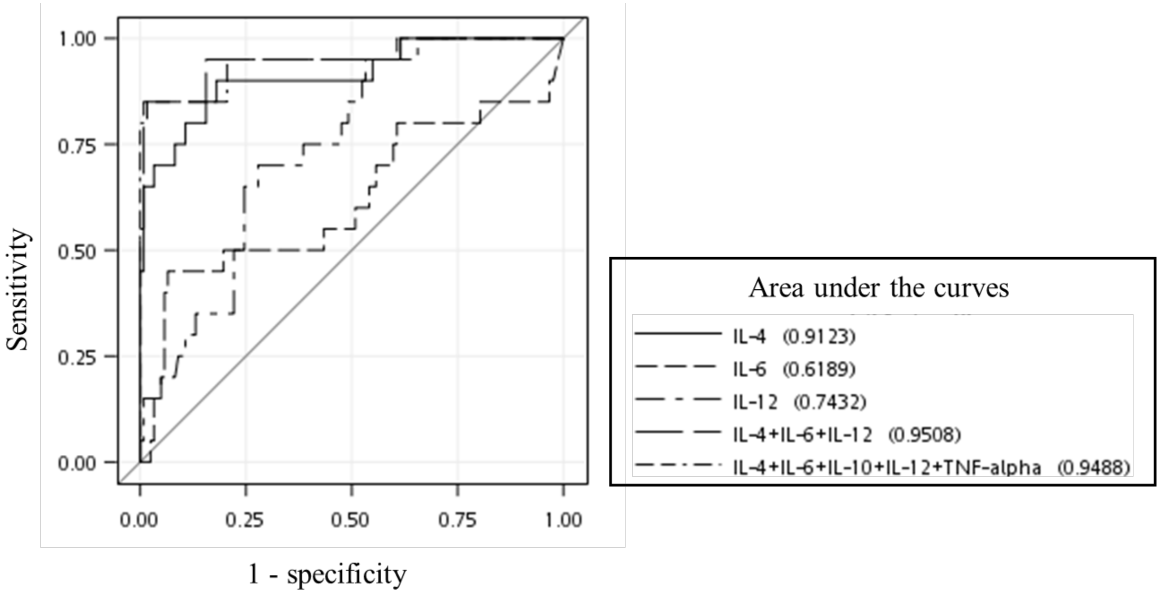

Figure S2. The receiver-operator characteristic (ROC) curves of plasma cytokines in early-onset schizophrenia with neurological soft signs (NSS).
